# Development of a Play-Based Motor Learning Approach (A.MO.GIOCO) in Children with Bilateral Cerebral Palsy: Theoretical Framework and Intervention Methodology

**DOI:** 10.3390/children11010127

**Published:** 2024-01-19

**Authors:** Maria Foscan, Antonella Luparia, Francesca Molteni, Elisa Bianchi, Shari Gandelli, Emanuela Pagliano, Ermellina Fedrizzi

**Affiliations:** 1 Fondazione IRCCS Istituto Neurologico C. Besta, 20133 Milano, Italy; maria.foscan@istituto-besta.it (M.F.); shari.gandelli@istituto-besta.it (S.G.); efedrizzi@libero.it (E.F.); 2 Istituto Neurologico Nazionale IRCCS Fondazione C. Mondino, 27100 Pavia, Italy; antonella.luparia@mondino.it; 3 Fondazione IRCCS E. Medea, 23842 Bosisio Parini, Italy; francesca.molteni@lanostrafamiglia.it; 4 Istituto di Ricerche Farmacologiche Mario Negri IRCCS, 20156 Milano, Italy; elisa.bianchi@marionegri.it

**Keywords:** Cerebral Palsy, rehabilitation, motor learning, children

## Abstract

The early intervention of motor training based on specific tasks and parent empowerment represents the new paradigm for the rehabilitation of children with Cerebral Palsy (CP). However, most published studies address the problem of the effectiveness of rehabilitation intervention without describing the treatment methodology or briefly mentioning it. The purpose of the study is to illustrate the development of a play-based motor learning approach titled A.MO.GIOCO (Apprendimento MOtorio nel GIOCO) and its systematization. Fifteen children aged between 2 and 6 years with bilateral CP will be enrolled and treated for 6–8 weeks (48 h). Motor Teaching methods applied by senior therapists have been extensively described, starting from rehabilitation goals and proposed therapeutic play activities, tailored to the functional profile of each child. This child-friendly rehabilitative approach (A.MO.GIOCO) refers to the systemic cognitive model of learning and movement control and is implemented in the context of spontaneous play activities and in the therapist–child–family interaction. In this study the theoretical framework of the approach and the process followed by the therapists to transfer it into rehabilitative practice are highlighted. As a result, an operational guide has been created. Further studies will explore the efficacy of the proposed standardized approach.

## 1. Introduction

Cerebral Palsy (CP) is defined as a group of permanent disorders concerning movement and posture development, which result in limited activities and can be attributed to the non-progressive permanent damage that occurred in the brain during fetal, neonatal, or infant development [1,2,3]. Motor disorders associated with infant CP are often accompanied by sensory, perceptual, cognitive, communicative, and behavioral disorders, epilepsy, and secondary musculoskeletal problems. The definition of Cerebral Palsy was published by Rosenbaum et al. in 2005 [4] following many consensus conferences of an international group of pediatric neurologists.

Bilateral clinical forms of spastic diplegia and tetraplegia, which are secondary to hemorrhagic and ischemic brain injury in preterm or term infants, have a prevalence of over 50% of cases of infant CP [5] and result in varying degrees of disability in both gross and fine motor skills. In recent years, global motor severity impairment has been classified using the Gross Motor Classification System (GMCS) [6], while for the fine motor severity impairment, the Manual Ability Classification System (MACS) has been used [7]. These gross and fine bilateral motor disorders are often associated with frequent neuro-visual disorders, including peripheral ophthalmologic and central disorders, such as Central Visual Impairment (CVI), which further affects the disability and quality of life of both the child and the family [8,9]. Visual impairment severity can be classified using the Visual Function Classification System (VFCS) [10].

As a result, in recent years, there has been a change in the cultural background of the rehabilitation approach, which in the past focused exclusively on motor aspects; therefore, the traditional rehabilitation methodology was represented by neuromuscular facilitation techniques (NDT therapy, such as Bobath, Vojta, and Doman) related to neuromaturation theories and based on the stimulation of reflexes or posture schemas, which, however, did not imply any motor learning. Novak reported the results of many publications, which confirmed the negative outcome of such treatment methodology [11]. Currently, motor functions are considered an expression of a perceptual–motor–cognitive process, which occurs in the search for a solution to a task that arises from the interaction between the individual and the environment. Therefore, CP rehabilitation is designed as a multidimensional approach based on systemic models of motor development and learning (the cognitive model of “Motor Learning”) [12,13,14].

The theoretical framework of this new rehabilitation approach, called the Motor Learning approach, represents a new paradigm based on information processing theories and supported by both clinical and instrumental data (such as functional Magnetic Resonance imaging, fMRI, and Diffusion Tensor Imaging, DTI). In fact, several cognitive psychologists, such as Bruner, Connolly, and Gibson [13,14,15], studying the development of child behavior during the first two years of life observed that all voluntary actions are based on a plan or abstract schema that lead the execution of motor programs, controlled at different levels of the sequence. Increasing the age, the child competence became greater and more adequate at the goal. In fact, the human brain comprises distributed cortical regions that are structurally and functionally connected into a network that is known as the human connectome. Elaborate developmental processes starting in utero herald connectome genesis, with dynamic changes in its architecture continuing throughout life [16]. The MRI data confirm the development of new connections during learning new competences and explain the Motor Learning process.

Recently, published studies [11,17,18,19] agree on emphasizing that the rehabilitative approach to children with CP should involve “goals-directed activity” in settings similar to the child’s living environment and should involve the family. In particular, Novak [17] highlights that recent randomized trials [18] indicate that an early motor training intervention based on specific tasks and with parental coaching represents the new paradigm for the rehabilitation of children with CP, since it promotes neuroplasticity and determines functional improvements. However, in general, most published studies address the problem of the effectiveness of rehabilitative intervention without describing the treatment methodology or even mentioning it briefly. The few studies that extensively report on treatment modalities include, for example, Charles and Gordon on HABIT [20] for children with hemiplegia and Morgan and Novak on the trial GAME [19]. It is considered essential not only to evaluate the effectiveness of treatment but also to spread its use among therapists, such that the approach be defined both in relation to theoretical reference models, the extent of functional impairment of the individual child, goals, settings, therapeutic proposals for activities, therapists implementation modalities, and transitions to the family environment.

It is with this objective that, since the 2000s, the Italian Cerebral Palsy Group (Gruppo Italiano Paralisi Cerebrale Infantile, GIPCI) has been organizing biennial training courses aimed for professionals involved in rehabilitation, in which, they have illustrated and discussed theoretical models regarding development and motor learning (motor learning and motor training), on which modern rehabilitative approaches are based. During these courses, videos of clinical cases with different forms of CP, evaluation methodologies of adaptive functions, and rehabilitative intervention according to the Motor Learning and Problem-Solving approach with “goals-directed activity” in play situations have been presented and illustrated.

In 2011, the GIPCI group published the results of a trial conducted on children with congenital hemiplegia, in which the outcome of two groups of children, one treated with Constraint-induced movement therapy (CIMT) and one with Intensive Bimanual treatment according to a play-based Motor Learning approach, was significantly better than the control group [21].

Playing, according to developmental psychology, represents a crucial role in learning and promotes both cognitive development and affective growth. In fact, Bruner [13] defines “motor game” during the early years of life, not as a repetition of the same patterns, but as the child’s continuous search for different solutions to a task that appears from the environmental interaction. Karmiloff-Smith [22] emphasizes the value of repeating a play sequence, which the child actively repeats for the pleasure gained from the progressive increase in their ability to obtain a result.

Symbolic and imitative play activities with rules are considered a means to create an atmosphere of understanding, harmony and shared pleasure, in which knowledge of reality is developed and the acquisition of motor, perceptual, praxis, and mnemonic skills is promoted. Play-based rehabilitation for children can therefore promote intentionality, creativity, knowledge, and pleasure, as occurs in psychomotor therapy, and provide an environment in which patterns and rules for solving tasks are exercised, thereby promoting motor learning and the development of more advanced skills.

**Figure 1:**
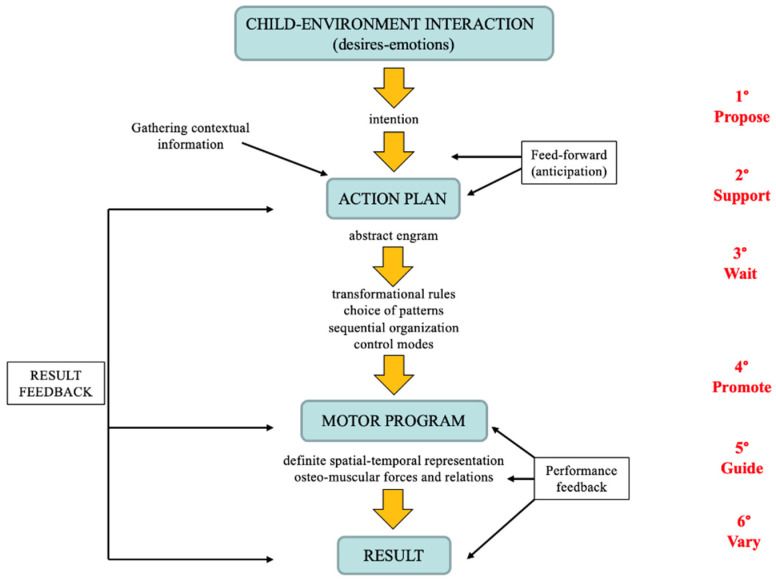
Levels of planning, execution, and motor behavior control according to the Motor Learning Model [23,24].

The setting of a spontaneous play program, in which tasks are natural and diversified and in which the child is the actor who develops hypotheses, analyzes environmental information, identifies strategies, and verifies results, as shown in Figure 1, represents the essential elements for a rehabilitative project that favors not only motor learning, but also the overall cognitive and affective development of the child with CP. The therapist will have to interact as a partner, supporting and helping the child in all phases of motor learning in resolving a task: the elaboration of an action plan, the collection of necessary information for defining a motor program, the explication of rules, the selection and control of sequences, the verification of results, and the possible re-elaboration of the plan or motor program.

This child-friendly rehabilitative approach presented here refers to the systemic cognitive model of learning and movement control and is implemented in the context of spontaneous play activities and in the therapist–child–family interaction. It was developed by professionals of the Rehabilitation Service of the Developmental Neurology Unit of the IRCCS Neurological Institute Carlo Besta in Milan and has been used for many years for the rehabilitation of children with CP [23,24].

The aim of the study is to illustrate the theoretical framework and to systematize the A.MO.GIOCO (Apprendimento MOtorio nel GIOCO) approach for children to make it homogeneous among therapists of the centers involved.

According to this aim, 15 children aged between 2 and 6 years with bilateral CP (GMFCS, MACS, and VFCS levels between II and IV) will be enrolled for the application of the A.MO.GIOCO approach, treated for 6–8 weeks (48 h), and compared to a control group, which will be treated with usual care and will be evaluated with the same protocol.

Patients will be assessed at baseline (T0), at the end of treatment (T1), and 6 months after the end of treatment (T2) using a protocol composed of the following standardized scales: Griffiths Scales of Child Development, Third Edition [25]; Gross Motor Function Measure scale (GMFM) [26]; Melbourne Assessment 2 scale (Ma2) [27]; Pediatric Evaluation of Disability Inventory (PEDI) [28]; LEA acuity tests, Visual Motor Integration [29]; Pediatric Quality of Life (PedsQL) [30]. A more complete description of the protocol will be provided elsewhere, as it is beyond the scope of this study.

## 2. Methods

### 2.1. The Rehabilitative Intervention: A.MO.GIOCO Approach

The A.MO.GIOCO approach, according to the Motor Learning Model explained in the Introduction, identifies different levels of intervention:

1. Propose to the child play situations and activities that evoke initiatives, desires for communication, and knowledge, all appropriate to their motor and cognitive abilities.
2. Support the child’s attention in formulating an action plan (analysis of information for the selection of strategies and tools, feedforward).
3. Wait for the child’s individual timing and initiative for the selection of motor scheme strategies and sequences, suitable for the implementation of the motor program.
4. Promote the motor program implementation with appropriate postures, stimulating visual and proprioceptive control and possibly facilitating the motor degrees of freedom control (performance feedback).
5. Guide the child in analyzing the results and any errors, both during the formulation of the action plan and execution of the motor program (outcome feedback).
6. Vary environmental settings to promote the use of new strategies and distinct motor programs.

Accordingly, to the different levels of interventions, therapists have to follow some fundamental rules in the rehabilitation intervention:

- The choice of the intervention goals must be based on the adaptive function profile that emerged during the evaluation, in relation to the individual child’s family and social environment, age, and level of cognitive and emotional development.
- To have therapeutic value, play proposals must always consider first and foremost the child’s motivation in carrying out the activity.
- The planning of the therapeutic intervention, and therefore its goals, setting, activity proposals, and problem-solving, must consider and integrate all aspects of the individual child’s functional disorder.
- The setting in which to insert play and activity proposals must be chosen in agreement with parents’ suggestions and must be as similar as possible to the family environment. The setting should also be various to promote the use of new strategies and diversified motor programs.
- During the rehabilitation intervention, the child should always be allowed and encouraged to take the initiative in choosing activities and planning their execution. The therapist must therefore be able to wait for the child’s individual timing and ways of choosing strategies and executing sequences, intervening, if necessary, with support and/or facilitation.
- During the evaluation phase and throughout all rehabilitation intervention phases, the therapist must always consider adaptive modifiability in the dynamic interaction with the child as an essential element for planning the therapeutic project. This requires the therapist’s flexibility and ability to modify proposals, settings, and play situations to reveal, during the interaction with the child, his/her most advanced skills in relation to their motivation and chosen activity goals.

### 2.2. Systematization of the Rehabilitative Intervention

Regarding the need to systematize the rehabilitative intervention, a first phase was carried out through discussions among senior therapists from the three centers involved, in order to delineate the principles of A.MO.GIOCO into concrete and shared rehabilitation proposals.

This required a long and diligent period of work, which was divided into several meetings between operators, both in person and remotely, to discuss the following:

1. Possible general goals for each main functional area (gross motor, manipulative, visual and visuo-cognitive);
2. How to pursue them based on the level of functional impairment of the child, classified according to GMFCS, MACS, and VFCS (levels II–IV);
3. Possible play and autonomy activities to propose.

Each senior therapist has drawn up the possible macro goals for one of the three main functional areas identified (gross motor, manipulative, visual, and visuo-cognitive), with the description of the type of materials/objects/furnishings to be used in the rehabilitation session based on the child’s functional level and age. The work produced individually was then reviewed by the other therapists for any corrections and additions and modified again until the final version was approved by all three therapists. Subsequently, examples of play activities in which to insert the goals were drawn up, starting from the analysis of video-recorded rehabilitation sessions in which game contexts were proposed to verify adherence to the rehabilitation objectives and the possibility of further modifying the proposal to better integrate all functional areas (gross motor, manipulative, visual, and visuo-cognitive).

Two main difficulties emerged: identifying precise macro-goals, but at the same time applicable to children with different levels of functioning and clearly highlighting the transition from goals to the rehabilitative proposal. The first aspect was addressed by structuring tables with reference to the possible classification level of each function, to be able to delineate the general objective in different settings and modalities based on the functions’ modifiability margin for that child. This structuring allowed for a general overview of goals and a very precise specification of the setting and modality created for each level of functioning. The second issue, i.e., the transformation of the goals into a concrete rehabilitative proposal, required a complex analysis and breakdown of the possible play activities, to produce a simple and clear underlying rationale. The section regarding the play activity examples was therefore accompanied by precise instructions, emphasizing how the operation of breaking down the proposal responds to a need for clarity of the therapist’s modus operandi and should not result in the rigid subdivision of activities to be proposed to the child. Given the need to standardize the intervention as much as possible, not only in the therapist’s mindset and methods, but also in the concrete activities proposed to the children, once these premises were defined, ample space was given to the numerous and various examples of play activities, which were described in detail, to provide multiple ideas to which the therapist can easily resort.

The Template for Intervention Description and Replication (TIDieR) checklist [31] was used to provide a comprehensive description of the intervention, consistent with CONSORT recommendations (Table 1).

## 3. Results

An operational guide has been created (see Table A1 and Table A2 for details) with instructions regarding the following:

1. **Therapists’ role (Table 2)**: methods that should be implemented by the therapist to support the child during all action phases, intervening as a guide at different levels, as described previously in this article.

2. **Macro intervention areas (Table 3)**: divided into two age groups, 2–4 years and 4–6 years, within which the possible main rehabilitative intervention goals were the focus, in reference to gross motor, manipulative-praxis, visual and visuo-cognitive adaptive functions. Materials and settings to be used during the preparation of the rehabilitative proposal were described for each goal, with a modification based on the different levels of motor, manipulation-praxis, and visual functionality obtained from international classification systems (GMFCS, MACS, VFCS).

For the gross motor area, this means, according to the terms used in the classifications, providing such a diversified setting:

For level II, a poorly adapted space, which provides positions and movements that are as variable as possible, in the presence of minimal external supports;

For level III it will be necessary to simplify the requests and provide a certain degree of external support, for example using surfaces or support bars, and aids, such as a walker;

For level IV the degree of postural containment, external stabilization, and external support through aids or other supports increases and the complexity of the request further decreases (for example, requesting trunk adjustments to reach an object while using a stander).

For the praxis manipulative area, the graduation of variability and complexity will be linked to the type of objects chosen, their mode of presentation, and the type of actions required, specifically as follows:

For level II, it will be possible to propose objects with different characteristics, trying to elicit grip and pattern adaptation and propose more complex activities with more subcomponents;

For level III, the objects must be easy to grasp, the actions required must have few subcomponents and it will be necessary to propose the objects in facilitating positions, for example frontally on the table;

For level IV, the objects will be very simplified, with characteristics adapted to the child’s grasping possibilities, simple actions, such as holding, releasing, and pressing, and constant support from the therapist will be necessary to complete the activity, as well as facilitating positioning of the object in space.

Finally, for the visual and visual cognitive area, the degree of adaptation to the environment will change in terms of visually adapted material, the distance at which to propose it, the action required, and the postural situation in which to present the activity, with examples as follows:

For level II, objects can be proposed without particular adaptations, possibly adapting the distance and speed of movement in visual tracking tasks; the proposals can be made both in a sitting position at the table and while the child is moving.

For Level III, the proposed material must be partially adapted, therefore with high visual contrast, with multisensory characteristics, of a larger size, reducing visual crowding, with supports, such as inclined planes at calibrated distances. Proposals will mostly be made while sitting at the table.

For Level IV, greater adaptations will be necessary, such as positioning illuminated objects in a semi-dark environment; the child will have to be constantly supported, and the use of other sensory modalities (touch, hearing) will also be encouraged.

Therefore, starting from the same goal, the proposal has to be outlined in different ways based on the level of the child’s functioning and therefore the need for less or greater environment adaptation and external support. The same child may have different levels of functioning depending on the area under examination, for example a level II in GMFCS but a level III in VFCS, and may therefore require little or no support from a postural point of view (child’s internal strategies to adapt to the environment will be mostly looked for), but an ad hoc adaptation of materials from a visual and visuo-cognitive point of view, for example simplified images, with good visual contrast and not too crowded.

3. **Examples of play settings (Table 4)**: Practical examples of motor, imitative-symbolic, constructive, graphic, and visuo-cognitive play proposals. Extensive space has been devoted to explaining how to “build” the activities, highlighting which parts of them lend themselves to the inclusion of goals for the different areas of intervention, hence gross motor, manipulative-praxis, and visuo-cognitive. The play activity proposal is subdivided into different sub-components and is not intended to be rigid, as it is not always possible to clearly separate the various functions since they are interconnected by nature; in some proposals, one of the three areas may be more represented than the other.

The goals that can be pursued within the game Treasure Hunt described in Table 4 are, for example, promoting postural transitions and movement in space, promoting monitoring and visual scanning both during movement and in searching for objects, promoting eye–hand coordination and orientation/calibration of the grip based on the characteristics of the object to be grasped and based on the shape of the container in which to insert it (for example for coins to insert into the piggy bank), and promoting visuospatial skills in translating movement on a map and vice versa in interpreting the coordinates of a map.

The purpose of the guide is to provide a mental reference guide, useful during the planning phase of the therapeutic proposal, to consider all aspects in play and “playable”. The proposals include starting points, which can be further developed based on the child to whom they are addressed. As already mentioned, the starting point for learning is the child’s motivation, which directs the action and also supports possible fatigue and frustration; therefore, the child’s play preferences, cognitive profile and emotional-affective, communicative, and relational characteristics should be firstly considered, thinking about who that child is at that moment in his or her path. These are the first elements that guide the therapist in choosing the play framework to propose, so that it is meaningful, motivating, and supports all areas of the child’s development.

Each play activity part will then be specified, in materials and settings, based on the child’s functioning level in that area, using Table A1; therefore, the same proposal can be made in different positions, with different containment degrees, in a static or dynamic situation, choosing the appropriate objects, their positioning, the type of visual clues, crowding, the use of aids or adaptations to the environment and to the objects, etc., based on what is specified for each area according to the classification level (GMFCS, MACS, and VFCS).

For example, for a 2-year-old child with GMFCS level III, miniMACS level II, and VFCS level III, the following macrogoals could be selected, based on the child’s functional assessment:

- Assess and promote methods of moving vertically with and without assistive devices in indoor environments (including transitions to and from the device).
- Encourage modulation and variability of reaching, grasping, and manipulation schemes in relation to the objects’ characteristics (size, shape, texture, orientation, weight).
- Promote visual scanning by favoring the use of a strategy aimed at organizing eye movements, initially in serial and then randomized settings, to support selective visual attention.

The context of the Treasure Hunt game can then be selected based on the child’s preferences, which will be declined in the following way, based on the rehabilitative goals and functional level of the child in the three areas.

A play space that is visually not too crowded will therefore be set up, with support surfaces or a parallel bar to allow walking with support. Ankle foot orthoses and shoes will possibly be used to promote ankle stabilization and a correct gait pattern. Medium-sized coins of different colors will be prepared to find and collect in an orderly manner along the route. The child will therefore have to, with the therapist, move by organizing the movement using the supports, visually monitoring the space, stopping and trying to position himself in a stable way to collect each coin along the way, put it in a bag by orienting his grip, and reach the end of the path to a series of small chests/containers of different sizes placed at an orderly distance in which to insert the coins won according to color to encourage visual scanning and analysis of the visual characteristics of the objects. Each container may have a different opening system to favor different practical patterns (unscrew, lift, insert).

Within this section, further indications are given regarding the inclusion of goals and proposals related to self-care autonomies, to be considered transversal, and they must always be shared with the family with the aim of supporting the child in developing their own skills within their daily environment and, at the same time, promoting parental skills and parenthood from the perspective of the transferability of skills learned during treatment sessions to the described life context.

It is important to include moments in which the children are accompanied in “doing things on their own”, based on possibilities and age, during activities, such as dressing, feeding, and hygiene: planning, useful strategies, using any possible aids or facilitations for object positioning and functional use (for example, modifications to utensils/glasses/plates or clothing with Velcro, magnets, elastic laces, etc.). Initial and final moments of the session can be used to accompany the child in dressing and undressing autonomously and possibly plan snack breaks to accompany feeding activities (for example, opening a package, fruit peeling, using a spoon to eat pudding, wiping with a napkin, pouring water into a glass, etc.) and if necessary, at the end, washing the hands and mouth. Furthermore, also during imitation play activities, it is possible to support the verbalization of sequences related to dressing/undressing a doll and subsequently apply them to daily life.

## 4. Discussion

This work illustrates the background and the systematization of a play-based Motor Learning rehabilitation approach titled A.MO.GIOCO, for children with bilateral forms of Cerebral Palsy.

In recent decades, particularly since the 2000s, several studies have been published regarding rehabilitative therapies based on Motor Learning and Motor Training principles for children with Cerebral Palsy (CP). Most of these studies are trials, which compare traditional therapy as neurodevelopmental therapy with new approaches originated from systemic theories [18,21,32]; on the other hand, there are fewer studies available that illustrate, in detail, the therapist’s intervention strategies [33] and proposals for appropriate goals based on the functional disorder type [20,34] and task characteristics and problems that the child must solve during the different stages of motor learning. The most important contribution in the delineation of Motor Teaching strategies by pediatric age was the model proposed by Larin [35], based on information processing theories, which emphasizes that the therapist must intervene with instructions before, during, and after the completion of the task proposed to the child, and it illustrates the essential elements for therapeutic practice: setting characteristics, motivation and significance of the task for that child, waiting for the child’s initiative, the demonstration or imitation of a problem-solving strategy, the guidance from the therapist, both the intrinsic and extrinsic reinforcement feedback, and the repetition of an activity.

Studies regarding the teaching modality of Motor Learning strategies by therapists involved in the rehabilitation of children with CP and measurement tools for the employment of such strategies have been recently published [33,36,37]. The Motor Learning Strategy Rating Instrument is a 20-item questionnaire that explores the ways in which therapists interact with children during physiotherapy sessions, evaluating “what the therapist says” and “what the therapist does” through video recordings, documenting and analyzing the Motor Learning content of physiotherapy interventions for children with CP. The use of this measurement tool has been found to be valid in increasing therapists’ awareness of their own ways of planning and conducting the decision-making project in relation to the child’s and setting characteristics. Another tool that measures the fidelity of pediatric rehabilitation, that is, the therapist’s adherence to the planned intervention, has been developed by the Canadian group CanChild [38] and named PROF (Pediatric Rehabilitation Observational Measure of Fidelity). With this tool, attributes of the therapist’s practice behavior in family-centered service are measured. Both tools have been developed to measure general attributes of the rehabilitation intervention, such as the therapist’s interaction with the child during treatment and the therapist’s adherence to the principles of Family Centered Therapy, whereas in the described A.MO.GIOCO approach, specific intervention attributes are illustrated.

The main contribution that this study made was the very positive appreciation from the therapists, even if this certainly involved long and complex work.

The result of this work, aimed at standardizing the rehabilitation treatment as much as possible, must be considered a guide that can never be exhaustive in relation to the complexity of the rehabilitation process for children with CP.

It can constitute a starting point that can be further defined more specifically based on the child and his characteristics. The chosen goals can only be general, applicable to all children in the sample examined but with necessary further specifications based on the individual child, which constitute the individualized and tailor-made characteristic of the rehabilitation intervention.

The experience of senior therapists who conducted the systematization of goals, materials, settings, and activities according to the A.MO.GIOCO approach allowed them to deepen their understanding in relation to Motor Teaching methods, specifically for the different goals identified in relation to the child’s characteristics (involved functional areas, disorder severity), for the age group, and for the possible presence of cognitive and visuoperceptual disorders. The theoretical framework knowledge gained through many years of experience has made it possible for them to make the clinical practice of Motor Teaching homogenous in the centers involved. The discussion, the comparison of experiences through videos, and the search for ways to transform theoretical rehabilitative projects into concrete operational proposals has given therapists awareness in relation to the decision-making process of which they were often unaware. Another important contribution of the A.MO.GIOCO rehabilitation approach systematization was the possibility of sharing with parents ideas of play activities that can be transferred also to the family setting in everyday life.

The study presented here provides an example of the declination of Motor Teaching and Motor Learning modalities and strategies, also delving into how these are applied in practice within the rehabilitation intervention with reference to rehabilitative goals/proposed game activities and differentiation based on the functional levels of the children. This important work of clarifying the rehabilitation approach has made it possible to make the proposed rehabilitation intervention clear and homogenous, so as to be able to rigorously verify its effectiveness compared to other approaches in a future study. Without this phase of description and standardization, the rehabilitation intervention would remain poorly defined and therefore verifiable.

In conclusion, as recommendations for future research, it is emphasized that in pa-pers on rehabilitation treatments, there is a need to further deepen the description of the theoretical models to which therapists refer, the methods they use to implement Motor Teaching strategies in their rehabilitation work, and the therapeutic goals.

## Figures and Tables

**Table 1:** TDieR checklist for intervention.

No.	Items	Details
1	Intervention name	A.MO.GIOCO (Apprendimento MOtorio nel GIOCO)
2	Why	The early intervention of motor training based on specific tasks and parent empowerment represents the new paradigm for the rehabilitation of children with Cerebral Palsy.
3	What	Goal-directed play and autonomy activities for children with bilateral CP aged 2–6 years, proposed according to motor learning and teaching principles (see Table A2 for details)
4	Who provided	Senior pediatric therapists
5	How	Face-to-face, individually, with the presence of the parent
6	Where	Rehabilitative centers
7	When and how much	6–8 weeks (48 h)
8	Tailoring	The starting point for learning is the child’s motivation, which directs the action and also supports possible fatigue and frustration; therefore, the child’s play preferences, cognitive profile, emotional-affective, communicative, and relational characteristics should be firstly considered. Materials and adaptations to be used during the preparation of the rehabilitative proposal were described for each objective, with a modification based on the different levels of motor, manipulation-praxis, and visual functionality obtained from international classification systems (GMFCS, MACS, VFCS) (see Table A1 for details).
9	Modification	The therapist must always consider adaptive modifiability in the dynamic interaction with the child. This requires the therapist’s flexibility and ability to modify proposals, settings, and play situations to reveal, during the interaction with the child, his/her most advanced skills in relation to their motivation and chosen activity goals.
10	How well planned for (adherence)	An operation guide has been created to assure adherence to the Motor Learning principles and to guide the therapist’s modus operandi. Practical examples of motor, imitative-symbolic, constructive, graphic, and visuo-cognitive play proposals have been included in the guide. Extensive space has been devoted to explaining how to “build” the activities, highlighting which parts of them lend themselves to the inclusion of goals for the different areas of intervention, hence gross motor, manipulative-praxis, and visuo-cognitive. Further indications are given regarding the inclusion of goals and proposals related to self-care autonomies, to be considered transversal and must always be shared with the family with the aim of, supporting the child in developing their own skills within their daily environment and, at the same time, promoting parental skills and parenthood for the perspective of the transferability of skills learned during treatment sessions to the described life context.

**Table 2:** Therapists’ role.

Levels of Intervention	Therapist Role
Setting	Propose activities that evoke intentions and desires for communication and knowledge, suitable for their profile, not only from a motor point of view but also cognitive, to their game phase and emotional and behavioral characteristics: In general, encourage environment and object exploration, interaction, communication, and the development of a game-action.
Action Plan	Encourage and support the child’s attention in creating an action plan (collection and analysis of perceptual aspects regarding the setting for the selection of executive strategies, tools, and means): For example, guide the child to observe relationships between objects and their position in space, direction, and distance to be covered, obstacles to be avoided, and the temporal sequence of the activity that must be carried out. Verbalize and/or ask the child to specify their goal and indicate the different phases of the execution sequence necessary to achieve the result (i.e., what must be done before and after).
Implementation and Control of Executive Sequences	Promote both the implementation and control of executive sequences: Wait for the child’s initiative and strategies and eventually facilitate the sequence executions with appropriate postures, prompts, and maneuvers. Encourage visual and proprioceptive monitoring during the implementation of such sequences, by also verbally supporting the child’s attention (external feedback).
Result Analysis	Guide the child in analyzing results and any possible errors, which could have occurred during the planning phase and the activity execution, with a gradual transition from external to internal feedback: Assist the child and evaluate whether the result corresponds to the goal of that game activity and support him/her in identifying any errors. Gradually reduce external control (verbalization and/or facilitation) and ask the child to verbalize or indicate the sequence during the execution.

**Table 3:** Example of goals and materials/setting for the age range of 2–4 years based on GMFCS of different levels.

Gross Motor Area			
Rehabilitation Goal	GMFCS II Materials/Setting	GMFCS III Materials/Setting	GMFCS IV Materials/Setting
Promote the transition to and from the upright position and intermediate positions (for example half-kneeling) in different settings, increasing the child’s awareness and autonomy.	Propose a play activity in which, for example, the following actions are required: - Transition from the ground to a half-kneeling position, standing position, and back, with support on the table, wall, parallel bars, walker. - Transition from the sitting position on the bench to the standing position and back, with support on the table, wall, parallel bars, or walker. - Standing up and sitting down from a roller or ride-on toy. Use ankle-foot orthoses or shoes if necessary.	Propose a play activity in which, for example, the following actions are required: - Transition from sitting position on a bench to a standing position and back, with a surface support, such as table, parallel bars, or a walker. - Standing up and sitting down from a roller or ride-on toy with support on parallel bars or a wall. - Transition from the ground to a kneeling position with anterior support on a bench. Activities in a tall-kneeling position. Use ankle-foot orthoses or shoes if necessary.	Propose a play activity in which the transition from bench to standing and back, using a bar for support and traction, is required.Use ankle-foot orthoses or shoes if necessary.

**Table 4:** Example of play activities.

Treasure Hunt		
Gross Motor Area	Manipulative Praxis Area	Visual and Visuo-Cognitive Area
Moving within an adventure path (horizontal displacement, independent walking, walking with a device).	Collect the hidden objects and put them in the treasure chest (which might have to be opened with a key) or find the doubloons and insert them into the piggy bank.	Follow footprints or trajectories on the floor. Optionally, introduce a map or therapist’s coordinates to follow during the activity (with older children), or ask the child to build the route using three-dimensional elements based on the therapist’s instructions to reach the finish line. Ask them to collect the doubloons or treasure objects arranged along the path in sequence.

## Data Availability

The data presented in this study are available on request from the corrisponding author. The data are not publicly available due to the enrolment and signing of the partecipants’ consent is in porgress, the content of our work is related to the methodology and we currently have no clinical data of the patients.

## Appendix A

**Table A1:** A.MO.GIOCO GUIDE: Macro areas of the intervention section (gross motor, manipulative-praxis, visual, and visuo-cognitive) with goals, materials, and settings for the age range 2–4 years based on different levels of the functional classification (GMFCS, MACS, and VFCS).

**Gross Motor Area**			
Rehabilitation goals	GMFCS II materials/setting	GMFCS III materials/setting	GMFCS IV materials/setting
Promote position changes and pre-locomotor movement schemes by facilitating dissociation between the girdles and trunk rotations, reactions in relation to support and balance, load shifting between the two hemisomes, alternating and rhythmic movement schemes between the two lower limbs.	Propose a play activity in which, for example, the following actions are required: in the pre-locomotor position, climbing over rolls, climbing up/down steps and soft wedges, tunnels, etc. Within the activity, the following can be proposed, according to the proposed game activity: objects to reach for/grasp/manipulate, etc., in different spaces.	Propose a play activity in which, for example, the following actions are required: in the pre-locomotor position, climbing over rolls, climbing up/down steps and soft wedges, facilitating changes in posture using support surfaces, etc. Within the activity, the following can be proposed, according to the proposed game activity: objects to reach for/grasp/manipulate, etc., in different spaces.	Propose a play activity in which, for example, the following actions are required: maintain a prone position with a small roll or low wedges positioned under the armpit to facilitate straightening and use of an upper limb during reaching activities, rolling/sliding using a low sledge to facilitate this. Within the activity the following can be proposed, according to the proposed game activity: objects to reach for/grasp/manipulate.
Improve trunk control and balance while seated in different and progressively more complex settings, integrating the use of the upper limbs.	Propose a play activity in which, for example, the following positions are required: on-the-floor sitting position with crossed legs, in long sitting, on the side, on a low wedge, on a stool with feet on the floor, astride on a roll or inflatable ride-on, etc. Within the activity, the following can be proposed, according to the proposed game activity: objects to reach for/grasp/manipulate, etc., in different spaces. Possible ankle foot orthoses.	Propose a play activity in which, for example, the following positions are required: on-the-floor sitting position with crossed legs, in long sitting, on the side, on a low wedge with a lateral and/or back support surface (for example, low roll). Sitting position on a bench with feet on the floor and variable trunk and pelvic containment/support. Sitting astride on a roll with feet on the floor. Activities can be proposed in all spaces when using a stool/bench or a roll, but also on an anterior vertical surface (i.e., mirror) or at a table (table with anterior hollow space). Within the activity, the following can be proposed, according to the proposed game activity: object to reach for/grasp/manipulate, etc. Possible ankle foot orthoses.	Propose a play activity in which, for example, the following positions are required: on-the-floor sitting position with a low wedge and support surfaces (i.e., lateral, or only posterior, angled), astride on a roll with pelvis stabilization and feet on the floor, possible anterior or vertical support surface or bar to hold onto. Sitting position with feet on the floor and a trunk and pelvic postural system with variable containment and support. Within the activity the following can be proposed, according to the proposed game activity: objects to reach for/touch (i.e., images in the mirror, bubbles to pop, simple effect games to press). Possible ankle foot orthoses.
Promote the transition to and from the upright position and intermediate positions (for example half-kneeling) in different settings, increasing the child’s awareness and autonomy.	Propose a play activity in which, for example, the following actions are required: - Transition from the ground to a half-kneeling position, standing position and back, with support on the table, wall, parallel bars, walker. - Transition from the sitting position on the bench to the standing position and back, with support on the table, wall, parallel bars, walker. - Standing up and sitting down from a roller or ride-on toy. Within the activity, the following can be proposed, according to the proposed game activity: object to reach for/grasp/manipulate/carry, etc. Possible ankle-foot orthoses or shoes if necessary.	Propose a play activity in which, for example, the following actions are required: - Transition from a sitting position on a bench to a standing position and back, with a surface support, such as table, parallel bars, or a walker. - Standing up and sitting down from a roller or ride-on toy with support on parallel bars or a wall. - Transition from the ground to a kneeling position with anterior support on a bench. Activities in a tall-kneeling position. Within the activity, the following can be proposed, according to the proposed game activity: object to reach for/grasp/manipulate/carry, etc. Possible ankle-foot orthoses or shoes if necessary; g position.	Propose a play activity in which the transition from bench to standing and back, using a bar for support and traction, is required. Within the activity, the following can be proposed, according to the proposed game activity: object to reach for/grasp/touch/carry, etc. Possible ankle-foot orthoses or shoes if necessary.
Promote the acquisition of the standing position (initially with bilateral support and gradually with the release of one or both upper limbs), with particular attention to alignment and weight distribution, and any perceptual component (with respect to the posterior space).	Propose a play activity in which, for example, the following positions are required: standing position with bilateral or unilateral support on surfaces/parallels/assistive devices/wall. Standing position with back support on walls or surfaces. Standing position without support. Within the activity, the following can be proposed, according to the proposed game activity: different surfaces to feel under the feet. Objects to indicate/touch/grasp/manipulate, etc., in different spaces. Possible ankle foot orthoses.	Propose a play activity in which, for example, the following positions are required: standing position with support on an anterior surface, on a walker or parallel bars, and possible posterior support on a wall. Within the activity, the following can be proposed, according to the proposed game activity: objects to indicate/touch/grasp/throw/place, etc., while freeing an upper limb. Possible ankle foot orthoses.	Propose a play activity in which, for example, the following positions are required: standing position with support on a bar or using an assistive device (stander). Encourage straightening and maintenance of the position, seeking trunk activation for small adjustments (i.e., using the upper limbs). Within the activity, the following can be proposed, according to the proposed game activity: objects to observe, touch, or use with the upper limbs while maintaining the erect position. Possible ankle foot orthoses.
Assess and promote methods of moving vertically with and without assistive devices in indoor environments (including transitions to and from the device).	Propose a play activity in which, for example, the following actions are required: cruising between surfaces at different heights, between two surfaces, on parallels, side-to-side with support on the wall or posterior support. Walking while pushing a wheeled toy, walking with a suitable assistive device. Within the activity, the following can be proposed, according to the proposed game activity: different surfaces to feel under the feet. Objects to indicate/touch/grasp/carry, etc., in different spaces. Possible ankle foot orthoses.	Propose a play activity in which, for example, the following actions are required: cruising, walking on parallel bars, walking with a suitable assistive device. Within the activity, the following can be proposed, according to the proposed game activity: different surfaces to feel under the feet. Objects to indicate/touch/grasp/carry, etc., in different spaces. Possible ankle foot orthoses.	Propose a play activity in which, the following actions are required: using the suitable assistive device (walker, wheelchair), explore the environment. Within the activity the following can be proposed, according to the proposed game activity: objects to indicate/touch/grasp/carry, etc., in different spaces. Possible ankle foot orthoses.
**Manipulative and Manual Praxis Area**			
Rehabilitation goals	MINI MACS II material/setting	MINI MACS III material/setting	MINI MACS IV material/setting
Encourage upper limb use for the following: - Communication (deictic and referential gestures); - Activities involving pointing, reaching, grasping, and moving objects.	Different objects/toys in terms of shape, size, texture, and weight; images/books to point at, reach, touch, grab, and move. Songs to mimic with gestures. Objects presented at all levels of the peripersonal space. Steady and contained sitting position: chair at an appropriate height for feet to rest on the floor (possible orthoses and shoes), table with a hollow space positioned at an appropriate height for comfortable support of the upper limbs. Less contained sitting position: sitting position on a bench with feet on the floor, lying down with the request of differentiating the use of upper limbs (one for load support activities and one for reaching and grasping activities), etc. Variable positions: standing, tall-kneeling. Dynamic situation: use of unstable/oscillating surfaces or during a movement.	Facilitating objects/toys: deformable, cylindric, with handles, web-like texture, easy to grasp (palmar grasp) and hold for minimal movement; balls/balloons to reach for and touch to induce movement, balls/toy cars to push (on a track or a mandatory path), soap bubbles to reach and pop, etc. Images/books on tangible material or on a PC (touchscreen) to facilitate pointing and reaching in the peripersonal space. Objects presented in an adapted position. Songs to mime with gestures. Constant support from the therapist to accompany sensory exploration and guide gestures. Sitting position on a suitable postural system with variable containment and support depending on the required effort. Adjustable table with a hollow space. Possible ankle foot orthoses and shoes.	Facilitating objects/toys: deformable, cylindrical, with handles, web-like texture, easy to grasp (palmar grasp) and hold for minimal movement; balls/balloons to reach for and touch to induce movement, balls/toy cars to push (on a track or a mandatory path), soap bubbles to reach and pop, etc. Images/books on tangible material or on a PC (touchscreen) to facilitate pointing and reaching in the peripersonal space. Objects presented in an adapted position. Songs to mime with gestures. Constant support from the therapist to accompany sensory exploration and guide gestures. Sitting position on an appropriate posture system with variable containment and support based on the required effort. Adjustable table with hollow space. Possible ankle foot orthoses and shoes.
Encourage modulation and variability of reaching, grasping, and manipulation schemes in relation to the objects’ characteristics (size, shape, texture, orientation, weight).	Variation in objects/toys in terms of shape, size, orientation, texture, and weight: spherical/cylindrical/cubic objects, plates; big/small, heavy/light; hard/soft; etc. Malleable materials (i.e., playdough, kinetic sand). Examples: common items, like bottle, containers, toy utensils/pots, toy cars, dolls/animals, construction block or games that must be separated/joined, markers (differentiate the use of both hands). Objects presented in all surfaces of the peripersonal space. Position/situation: as for the previous goal.	Objects/toys that adapt to the hand in terms of shape, size, orientation, consistency, and weight: cylindrical, spherical/cubic of a medium size, soft, and deformable, etc. Malleable material (for example: playdough, kinetic sand). Possible adapted grips. Examples: common objects, such as bottles, containers, toy utensils/pots, toy cars, dolls/animals, construction block or games that must be separated/joined, markers to encourage the differentiated use of the hands (one hand holds while the other explores the game). To facilitate task execution, the therapist can stabilize the limb, and the object can be stabilized against the support surface or against the trunk. Objects presented frontally, in a facilitated position. Position/situation: as for the previous goal.	Objects/toys that adapt to the hand and malleable unstructured material. Soft, deformable, cylindrical objects, with rings/handholds, web-like texture. Examples: kinetic sand, playdough, colorful carnival steamers, various types of paper, balls/toy cars, etc. Possible adapted grips. Objects presented in an adapted position. Constant support from the therapist to accompany sensory exploration and guide gestures. Position/situation: as for the previous goal.
Promote the acquisition and refinement of unimanual and bimanual praxis with gradual praxis progression (intrinsic synergies).	Objects/toys that involve actions, such as pushing, pressing buttons, pulling levers, rotating, striking, fitting into slots, etc. Objects of different sizes to be threaded onto different supports (from rigid ones—sticks, malleable plastic wire—to soft ones—string with initial wooden part); objects to be separated and joined (magnetic objects, with Velcro); construction blocks/Lego/different sizes of blocks to be stacked, lined up, joined, and separated; containers of different sizes to be opened/closed, in which small objects can be inserted; different types of paper to tear, fold, cut, crumple; unstructured material to mix, transfer, shape (flour, sand, etc.), pens to open/close, common objects or toys, such as tools/pots to manipulate by differentiating the use of both hands. Objects presented in all surfaces. Position/situation: as for the previous objective.	Objects/toys that involve actions, such as pushing, pressing buttons, pulling levers, picking up, and carrying; material to be inserted into different rigid supports (sticks, malleable plastic wire, thick wire with a rigid initial part), different sizes of magnetic objects or Lego to build small constructions, paper to tear and fold. Objects presented in a facilitated position, facing the individual. Position/situation: as for the previous objective.	Objects/toys that involve actions, such as pushing, pressing buttons, pulling levers, rotating, and smashing. Objects presented in an adapted position. Constant support from the therapist to accompany sensory exploration and guide gestures. Position/situation: as for the previous objective.
Promote praxis-manipulative organization through constructive activities, stabilizing the dominance and functional use of a writing tool.	Activities to support simple bimanual manipulative synergies: making puzzles up to eight pieces, building Lego constructions using a model and paper. Initial use of the upper limbs for differentiated activities in support of defining the dominance (one hand reaches, touches, and takes, while the other holds the support). Path executions using a writing tool with a progressive increase in difficulty (reduction in the size of the path guide, increase in the complexity of the path). Objects presented on all surfaces. Position/situation: as for previous objective.	Activities to support simple bimanual manipulative synergies: making puzzles up to eight pieces, building constructions with magnetic or Velcro material, using models. Initial use of the upper limbs for differentiated activities in support of defining the dominance: one hand reaches, touches, and takes, while the other maintains support. Path executions using a writing tool (possible small aids/adaptations) with the introduction of executive facilitations, fixing the paper to the support surface. Objects presented frontally, in a facilitated position. Position/situation: as for the previous objective.	Activities to support the differentiation of dominance using simple tasks (one hand reaches, touches, while the other maintains) with executive facilitations/use of technological tools (PC with touchscreen, tablet, trackball). Objects presented in an adapted position. Constant support from the therapist to accompany sensorial exploration and guide gestures. Position/situation: same as previous objective.
**Visual and Visuo-Cognitive Area**			
Rehabilitation goals	VFCS II material/setting	VFCS III material/setting	VFCS IV material/setting
Promote attentional aspects by involving the visual channel in terms of alertness and vigilance, promote oculomotor skills to stabilize and provide a lasting visual fixation, broaden, and provide fluid eye movements in different gaze directions (horizontal, vertical, diagonal, circular). NOTE Dynamic situation: during movement/in an upright position with any aids/orthoses/equipment if necessary. Static situation: sitting at a table with possible aids/orthoses.	Dynamic situation examples: Activities with marbles, toy cars, trains, spring toys, or rechargeable animal toys that move along a path with simple trajectories, which then become more complex, gradually increasing the distance. Static situation examples: Wooden labyrinths or magnetic boards on which magnets can be slid along linear, horizontal, vertical, circular, or diagonal trajectories within interactive visual scenes. Visual localization activities: identifying target objects in different sectors of the proximal space. Materials that can be used: three-dimensional objects/toys. Possible use of software for PC and/or tablet.	Dynamic situation examples: Activities with marbles, toy cars, trains, spring toys, or rechargeable animal toys that move along a path with simple trajectories, which gradually become more complex, at different distances on visually adapted support surfaces (structured with high chromatic contrast, such as a chessboard) or surfaces with contrasting colors to the selected toy (i.e., a white ball on a red surface). Static situation examples: Wooden labyrinths or magnetic boards on which magnets can be slid along linear trajectories, initially horizontal and vertical, and subsequently, circular or diagonal within visually interactive scenes of a simple configuration and contrasting colors with the targets (i.e., moving the red car on a chessboard track within the red box, moving the yellow car within the yellow box). Visual localization activities: identifying target objects in different sectors of the proximal space. Materials that can be used: visually adapted with high chromatic contrast, with possible multisensory information integration. Possible use of aids, such as a bookstand/inclined surface and software for PC and/or tablet.	Static situation examples: Using luminous targets and/or medium-sized structured targets with high chromatic contrast (i.e., 30 × 50 cm optical panels with checkerboard, polka dots, stripes) to be slowly transported on the horizontal and vertical surface at a close distance to the child’s face; using sound-tactile integration in situations where visual engagement is less easily evoked or less constant; move objects that produce sound on luminous surfaces or structured panels with high visuo-tactile contrast (i.e., sound balls on illuminated surfaces (“light box”) or checkerboards with embossed colors); cause–effect activities using software (i.e., monitor color change during contact) to which sound information can also be associated; visual localization games using two medium–large objects with multisensory characteristics, alternating them between frontal and sagittal positions. Materials that can be used: highly visually adapted (illuminable) with multisensory characteristics. The use of aids, such as a bookstand/inclined surface and use of software for PC and/or tablet may be necessary.
Promote visual scanning by favoring the use of a strategy aimed at organizing eye movements, initially in serial and then randomized settings, to support selective visual attention. NOTE Dynamic situation: pre-locomotor patterns/standing with the possibility of using any aids/orthoses/equipment. Static situation: sitting at a table with any aids/orthoses.	Dynamic situation examples: Activities that involve visually searching for objects by color and/or shape and/or size and/or semantic images, following an agreed order within an organized spatial situation following a psychomotor course trajectory, with increasing levels of difficulty correlated with the progressive and gradual increase in the number of objects/images and trajectories. Static situation examples: Activities that involve visually searching for objects, upon request/guidance, by color and/or shape and/or size and/or semantic images, initially arranged in sequence within a circumscribed space and subsequently in a scattered order, with increasing levels of difficulty correlated with the number of objects. Making the child visually search for details of an object and/or image in visual comparison tasks between similar objects and/or images, with increasing levels of difficulty correlated with the progressive increase in number and richness of details. Materials that can be used: three-dimensional/two-dimensional objects/games, possible use of software for PC and/or tablet.	Dynamic situation examples: Activities that involve visually searching for objects by color and/or shape and/or size and/or semantic images, following an agreed order within an organized spatial situation following a simple psychomotor course trajectory while maintaining high chromatic contrasts between objects and the object environment, with increasing levels of difficulty correlated with the progressive and gradual increase in the number of objects/images and trajectories (i.e., arranging objects on a checkerboard background). Static situation examples: Activities that involve visually searching for objects, upon request/guidance, by color and/or shape and/or size and/or semantic images, initially arranged in sequence within a circumscribed space and subsequently in a scattered order on backgrounds with contrasting chromatic colors to objects; increasing levels of difficulty correlated with the number of objects. Making the child visually search for details of an object and/or image in visual comparison tasks between similar objects and/or images, with increasing levels of difficulty correlated with the progressive increase in number and richness of details, which may have multisensory characteristics (i.e., associating an element—color—with a tactile variable—wool texture). Materials that can be used: perceptually visually adapted (structured with high chromatic contrast with possible integration of a sound–tactile information); use of aids, such as a bookstand/inclined surface; use of software for PC and/or tablet.	Static situation examples: Activities that require visually searching for a few illuminated objects in a graphically simple interactive scene (i.e., landscapes drawn on illuminated materials, like plexiglass); activities that require visually searching for details in a visuo, sound–tactile object/image (i.e., multisensory books: the child is encouraged to find visual, tactile, and/or sound details, such as “where is the dog’s tail?” which would be made of soft fabric); visual comparison tasks between two similar objects/images, which have few similar visuo-sound-tactile details; the same activity will be repeated with increasing difficulty, for example, with a progressive increase in the number of objects/images and details. Materials that can be used: highly perceptually adapted (illuminable) with multisensory characteristics, use of aids, such as a bookstand/inclined surface, use of software for PC and/or tablet.
Promote oculomanual coordination and visuomotor and visuo-graphomotor integration, to encourage the visual monitoring of actions and support attention and visuo-spatial perception processes; the visuo-spatial aspects will involve the following: (a) analysis of specific visuo-spatial configurations; (b) promotion of spatial perception processes (allocentric vs. egocentric); (c) promotion of object mental representation processes. NOTE Dynamic situation: kneeling/standing/moving with possible aids/orthoses/equipment. Static situation: sitting at a table with possible aids/orthoses.	Dynamic situation examples: Aiming at a target placed at a distance in the child’s peripersonal space with increasing levels of difficulty as the number of balls/bags and distance between the child and the target increases. Static situation examples: Stacking construction blocks, threading beads after visually searching for objects to be used based on shape, color, and size with increasing levels of difficulty related to the number and association of shape/color/size. Reproducing three-dimensional and two-dimensional models, where the goal is to maintain correct orientation, spatial relationships, and color as in the model; visually remembering the spatial location of objects within a grid and/or graph paper according to the initially offered model, with increasing levels of difficulty related to the progressive increase in the number of objects; first graphic activities following horizontal and vertical trajectories. Materials that can be used: three-dimensional and two-dimensional games, and possible use of software for PC and/or tablets.	Dynamic situation examples: Aim at a target placed at a close distance in the child’s peripersonal space in a visually adapted environment (i.e., using high chromatic contrast backgrounds) with increasing levels of difficulty for the progressive increase in number of balls/bags and distance between the child and the target. Static situation examples: Stacking/threading a few blocks/beads after visually searching for objects to be used according to shape, color, and size, with increasing levels of difficulty correlated with the number and association of shape/color/size; reproducing two-dimensional and three-dimensional models where the goal is to maintain the correct orientation, spatial relationships, and color as in the model; visually recalling the spatial placement of objects inside a grid and/or grid paper following the initially offered model, with increasing levels of difficulty correlated with the progressive increase in the number of objects; early graphic activities following horizontal and vertical trajectories on surfaces with different inclinations. Materials that can be used: perceptually visually adapted (structured with high chromatic contrast with possible audio-tactile information integration); use of aids, such as a bookstand/tilted surface; use of software for PC and/or tablet.	Static situation examples: Finding multisensory objects in different sectors of space (above/below, right/left) placed on magnetic boards or on support surfaces covered with Velcro with contrasting colors compared to the objects used; stacking few cubes after visually and tactually searching for them by shape, color, texture, size; reproducing three-dimensional and two-dimensional models while maintaining correct spatial relationships based on visuo-tactile information (color and texture), using a limited number of pieces; remembering the spatial location of a few visuo-tactile objects within a grid/form by inviting the child to firstly touch and subsequently reproduce the model. Materials that can be used: highly visually adapted (illuminable) with multisensory characteristics; use of aids, such as a bookstand/inclined surface, possible use of software for PC and/or tablet.
Promote visual analysis and identification processes of perceptual elements, such as color, shape, and orientation, for the visual recognition of objects/faces in different and gradually crowded settings of increasing difficulty. NOTE Static situation: sitting at a table with possible aids/orthoses.	Static situation examples: Matching games in terms of shape, color, size, and function (early semantic categorization); games in which the child must find identical objects within an interactive visual scene; image reconstructions (i.e., human or animal faces) with increasing difficulty levels as the number of puzzle pieces and complexity of details increase. Materials that can be used: three-dimensional games and the possible use of software for PC and/or tablets.	Static situation examples: Matching games in terms of shape, color, size, and function (early semantic categorizations); games in which the child must find identical objects within an interactive visual scene; reconstruction of images with increasing difficulty levels as the number of pieces and complexity of details increases. Materials that can be used: perceptually visually adapted (structured with high chromatic contrast with the possible integration of sound–tactile information); use of aids, such as a bookstand/inclined surface; possible use of software for PC and/or tablet.	Static situation examples: Matching games for shape, color, size, and function (early semantic categorizations) while also incorporating visuo-tactile characteristics (texture); games in which the goal is to find matching objects within an interactive visual scene with visuo-tactile embossed images; image reconstructions with increasing levels of difficulty as the number of pieces and complexity of detail increases. Materials that can be used: highly perceptually adapted (illuminable) with multisensory characteristics; the use of aids, such as a bookstand/inclined surface; as well as software for PC and/or tablet.

**Table A2:** A.MO.GIOCO GUIDE Example of play activities and instructions section. A.MO.GIOCO GUIDE.

Instructions		
In the first column, the play activity section, which lends itself to the inclusion of the gross-motor area goals is presented; the second column presents the play activity section that lends itself to the inclusion of goals regarding the manipulative-praxis area; lastly, the third column presents the part of the play activity, which lends itself to the inclusion of visual-visuo-cognitive area goals. It should be emphasized that the division of each area is not intended to be rigid, since it is not always possible to clearly differentiate the various functions at play, as they are interconnected by nature; moreover, in some proposals, one of the three areas may be represented more compared to the others. Such a schema simply provides a mental reference guide, useful for considering all aspects at play and “playable” during the therapeutic proposal planning phase. Since it is considered transversal; the self-care autonomies area is deliberately separated from the others. The proposals establish starting points, which can be further developed based on the child to whom they are addressed (level and game preferences, motivation). Each play activity part will be specified, within the materials and setting, based on the child’s level of functioning in that area; hence, the same proposal can be made in different positions, with different degrees of containment, in static or dynamic situations, choosing suitable objects, their positioning in space, the type of visual clues, crowding, the use of aids or adaptations to the environment and to objects, etc., according to what is specified for each area based on the classification level (GMFCM, MACS e VFCS).		
Motor Game Examples		
Gross Motor Area	Manipulative Praxis Area	Visual and Visuo-Cognitive Area
Treasure Hunt		
Moving within an adventure path (horizontal displacement, independent walking, walking with device).	Collect the hidden objects and put them in the treasure chest (which might have to be opened with a key) or find the doubloons and insert them into the piggy bank.	Follow footprints or trajectories on the floor. Optionally, introduce a map or therapist’s coordinates to follow during the activity (with older children), or ask the child to build the route using three-dimensional elements based on the therapist’s instructions to reach the finish line. Ask them to collect the doubloons or treasure objects arranged along the path in sequence.
Tag		
In a static situation (sitting on the floor or on a bench, tall-kneeling, half-kneeling, or standing position) or in a dynamic situation where a change in position is required (i.e., standing up from a half-kneeling position). Imitate fishing on a boat, a shopkeeper setting up a stall, or a collector cataloguing their objects.	Collect toy fish scattered throughout the surrounding space using a stick/net/magnetized fishing pole and put them in a basket. Attach/detach objects (fruit, balls, etc.) with Velcro/magnet/suction cups to a mirror/wall. Use an upper limb to lean (on a surface, on the mirror, on a handle) while the contralateral limb is engaged in grasping, etc.	Search, among many objects, for items to collect based on the request, such as fruit to sell, balls of a specific color, or other objects to create a collection. Then arrange the objects in a defined sequence (i.e., in a line or column, by category, etc.) or arrange the objects in boxes or inside circles of the same color.
Animal or Position Mimes		
On the floor, imitate the animals’ gait (i.e., crawling like a cat, waddling on knees like a penguin, slithering like a snake or caterpillar, rotating on the belly like a turtle, etc.) or while walking (i.e., taking long or short strides like a stork or an ant, walking fast or slow like a hare or a snail, etc.). Mimic the position of the child depicted in the chosen picture.	Take the selected animal, transport it, and position it in its paddock. Mimic the position of the child depicted in the chosen picture.	Select the animal to imitate by choosing it from pictures or stuffed toys. Optionally, introduce trajectories to follow during the movement. Finally, position the chosen animal in the correct house, associating it with the corresponding family. Mimic the position of the child depicted in the chosen picture, verbalizing it (spatial references in particular) with the therapists’ help.
Music Game		
Following the music rhythm, move on the ground or while walking, with or without an assistive device (i.e., slow-fast, stop-start), stand up and sit down according to the music volume, lean sideways, march in place, etc., based on the rhythm or words of a song.	Optionally, the child can hold and play a musical instrument, such as a tambourine or maracas. Mimic the gestures of a song (i.e., wave, point, etc.).	Optionally, move within simple trajectories.
Dice and Pawn Game		
Walk on colored tiles taking the number of steps indicated by the just-rolled dice, race with the therapist to see who gets to the end of the route first.	Grab the dice with both hands and roll it.	Together with the therapist, count the result of the dice roll, by counting the dots on the dice. With the therapist, monitor and count the number of steps/tiles to take. With older children, you can also add right/left forward/backward movements.
Footprints Game		
Walk to leave footprints after coloring or dipping them in talcum powder.	Take off and put on shoes, socks, or orthoses. At the end of the game, use a towel to clean the feet.	Monitor the footprints left, following simple trajectories.
Swords Game		
In a standing position, without leaving one’s own area (circle, colored tile, etc.), engage in swordplay with the therapist, inducing load transfers between the two hemisomes, anterior–posterior (i.e., take a lateral step or a knights’ lunge).	Maintain the sword grip, possibly also introduce a shield to hold on using the contralateral hand and control the upper limb movement to strike the opponent and defend oneself.	Monitor the area in which one can move to avoid stepping out of the designated game zone. You could also keep score by using a whiteboard, for example, placing magnets of different colors in two columns, one for each player, and at the end of the game, count the number of wins for each player (a magnet for every won game).
The Target Game		
In different postural positions (sitting down, tall-kneeling, or standing with or without support/assistive device), play by throwing towards a target or goal. Kick a ball from a still position with different degrees of support.	Using hands, grab and throw or roll a ball, a marble, or a bag in a box to score a basket, to reach a target, to knock down bowling pins, or push a toy car to simulate a race. Press the toy car or the button to move it along a path.	Within a circumscribed space, identify the target to hit (based on the therapist’s instructions) or maintain the trajectory of the marble or toy car within a track, or throw the ball/bag into the correct box (i.e., the color of the object).
Symbolic and Imitative Play Activities Examples		
Role-Playing Game		
Move by crawling or in standing position, with or without assistive device, maintain a prolonged standing position with or without assistive device, to prepare the various zones, and transport all useful objects for the game. For example, prepare the table, prepare the sleep/bath/doctor zone, carrying animals or dolls from one area to another, carrying cars from one area (parking lot) to another (garage, gas station, mechanic). Walk on different height levels with stop and go movements to carry food, like a waiter or grocery shopping. Act as a cashier in an upright position, with or without support or aid (static).	Using grocery items (plastic food, malleable dough, cereals, other manipulative materials) and various dishes/containers to prepare baby food (pouring, mixing, breaking, cutting, opening/closing containers, etc.); using doctor’s tools to examine dolls/animals; using mechanic’s tools to fix toy cars; paying for groceries by inserting coins into the cash register, etc. Dressing up as a doctor/mechanic/chef, etc. (using appropriate clothing with buttons, zippers, etc.). Dressing and undressing dolls/toys according to the game, using clothing with buttons, zippers, strings, etc.	Moving around a defined space, possibly following a path, reaching different food items or products to buy, recognizing, and choosing based on a list/recipe. Collecting the items in sequence along the path. Finally, arranging the items in a defined order, for example, playing the role of a fruit seller who arranges fruit crates based on specific instructions (above/below/in front, etc.). Same thing for toy cars in garages, etc.
Pilot Game		
Astride on a roller, sitting or standing on rocking surfaces, tilting, or rotating from side to side pretending to drive a motorcycle, pirate ship, etc., or standing up and sitting down to operate the controls of a spaceship, pirate ship or car, etc.	Maintain and direct a hoop or steering wheel (either placed on a surface or held), reach and press images or suction cups attached to the mirror or front wall to activate the controls of a spaceship, pirate ship, car, etc.	Explore the peripersonal space to locate the command to be executed according to the request.
Constructive Games, Expressive-Graphic, and Visuo-Cognitive Activity Examples		
Architect Game		
In a sitting position with or without support, in a stander device with a table in front.	Grasping, orienting, and positioning nails, pictures, sticks, tangrams, Legos, beads, cubes, etc. Using a PC or tablet, select with a finger on a touchscreen or use a keyboard/switch/joystick.	Reproduce the sequence of nails or beads while respecting the order. Reproduce a model to recreate a construction, a path, a scene, etc., in two or three dimensions.
Detective Game		
In sitting position with or without support, in a stander device with front table. For more skilled children, it can also be performed standing with a sheet attached to a mirror/wall.	Circle/mark/check the target element using a marker, a stamp, or a finger dipped in color. Use a PC or tablet to select with a finger on a touchscreen or use a keyboard/switch/joystick.	Provide multiple images of an object and find the individual details that differ or are missing. Alternatively, find identical images in a group. Alternatively, explore a sequence of images in order and find the target figure, or find a shape within an image or recognize two identical meaningless figures.
Theatre Game		
In a sitting position with or without support, in a stander device with a front table.	Grasping puppets/cutouts and moving them by inserting them into the mini environment holes. Optionally, move metallic elements with a magnetized support.	Make puppets or shapes appear/disappear from holes of a cardboard mini environment, animating the narration of a fairytale. Move metallic elements with a magnet, following their movement in the scenario according to the story.
Cards/Board Game		
This can be performed in a sitting position with or without support, in a stander device with a front table. Skilled children can also stand up and have an anterior surface. The activity can also be inserted into a simple path (i.e., completing a path to collect materials that will then be used in the game, or initially choosing a target—i.e., drawing a card from a deck—completing a short path to reach the table area where the game is completed by finding the matching card among many arranged in a row, etc.).	Using cards, bingo, tiles. Using a PC or tablet, select with your finger on the touchscreen or use a keyboard/switch/joystick.	Recognition and association of images in terms of the shape, color, dimension, function, and semantic category.
Domino Game		
This can be performed in a sitting position with or without support, in a stander device with a front table. Skilled children can also stand up and have an anterior surface. The activity can also be inserted into a simple path (i.e., completing a path to collect materials that will then be used in the game, or initially choosing a target—i.e., drawing a card from a deck—completing a short path to reach the table area where the game is completed by finding the matching card among many arranged in a row, etc.).	Grasp tiles and align them. Use PC or tablet to select with the finger on touchscreen or use keyboard/switch/joystick.	Explore and select correct images, arrange them in a row according to the rules of the game (such as the same shape or semantic category).
Position Game		
In sitting position with or without support, in a stander device with a front table.	Grasp and move the puppet on the grid. Peel and stick stickers on the grid. Stamp on the correct square. Use a PC or tablet to select with a finger on the touchscreen or use a keyboard/switch/joystick.	Move the puppet, the subject of the story, within a grid of squares according to the spatial positions indicated by the therapist to reach the goal of the path: “take two steps to the right and then continue for three steps forward.” Reproduce, with or without a model, the element arrangement on a grid or grid-lined sheet (i.e., dots, small characters, stamps).
Dice and Form Game		
In sitting position with or without support, in a stander device with a front table.	Taking turns with the therapist, pick up and roll the dice. Pick up the shape drawn on the face of the dice that comes up, choosing it from within a row.	Monitor the roll of the dice, systematically explore the row of shapes, and select the one corresponding to the drawing on the face of the dice.
Trace Game		
In sitting position with or without support, in a stander device with a front table.	To connect two elements, trace a trajectory in sand/flour/etc., using your finger. Use different graphic tools to complete linear or circular paths (cards). Use a computer or tablet to use your finger on the touchscreen or use a keyboard/switch/joystick.	To connect two elements, follow a path with your finger, or draw a line within linear or circular paths (the child to the house, etc.).
Artist Game		
In a sitting position with or without support, in front of a table or mirror or wall, or on a stander with a frontally inclined surface. For more skilled children, standing with a frontal surface may also be possible.	Draw, cut, paste, make collages, paint on a mirror, wall, or inclined surface. Choose the appropriate graphic tool or use your finger, hands, or PC/tablet, tracing directly on the touchscreen.	Visually monitor the graphic gesture, possibly following the therapist’s instructions, on the areas of the sheet to be filled, tracing lines within trajectories, coloring within the outlines of a drawn figure, etc.
